# Ultrasonic Assessment of the Influence of Cold Rolling and Recrystallization Annealing on the Elastic Constants in a TWIP Steel

**DOI:** 10.3390/ma14216559

**Published:** 2021-11-01

**Authors:** Linton Carvajal, María Sosa, Alfredo Artigas, Nelson Luco, Alberto Monsalve

**Affiliations:** Department of Metallurgical Engineering, Universidad de Santiago de Chile, Av. Ecuador 3735, Estación Central, Santiago 9170124, Chile; maria.sosa.o@usach.cl (M.S.); alfredo.artigas@usach.cl (A.A.); nelson.luco@usach.cl (N.L.); alberto.monsalve@usach.cl (A.M.)

**Keywords:** ultrasound, TWIP steel, elastic constants, Poisson’s ratio, birefringence, cold rolling, recrystallization

## Abstract

The evolution of the elastic constants, $C_{33}$, $C_{44}$ and $C_{55},$ Poisson’s ratio and acoustic birefringence of a Fe-0.5 wt% C-21.5 wt% Mn twinning-induced plasticity (TWIP) steel with reduction by cold rolling and recrystallization annealing was assessed from measurements of the times of flight of ultrasonic waves propagating along the thickness of the rolled plates. As the reduction increased, changes in the elastic constants resulted in a steadily increasing orthotropy, which was clearly shown by Poisson’s ratio and acoustic birefringence. Although optical metallography and hardness measurements showed that partial or full recrystallization is attained after annealing at 600 °C and 700 °C, the ultrasonic measurements revealed that a high level of orthotropy remains.

## 1. Introduction

Twinning-induced plasticity (TWIP) steels are currently among the most attractive materials for structural applications in the automotive industry, shipbuilding, oil and gas exploration and nonmagnetic structural applications [1,2]. This is due to their high strain hardening and high levels of fracture strength, a unique combination of strength and ductility that has been the focus of research and growing scientific and technological interest in recent years [3,4,5]. These steels present a full austenitic microstructure, which is stable at room temperature because of the high content of manganese and other alloying elements, which allow them not to undergo phase transformations during processing or cooling. Additionally, those alloying elements affect the stacking fault energy (SFE) of TWIP steels [6,7], which, as reported by De Cooman and Jung [8], lies between 20 and 40 mJm^−2^. Several authors have reported that even though deformation in TWIP steels is initiated by gliding dislocations, twinning has an unquestionable role in the development of high strain hardening. This mechanism of strain hardening is related to an interaction between dislocation glide and twinning that restricts the dislocation mean free path. Discontinuous serrated curves have been found in these steels at room temperature [9], being related to dynamic strain aging. Additionally, TWIP steels are characterized by their high impact energy and fracture toughness [10].

There is consensus on the fact that cold rolling induces in TWIP steels what can be described as a brass-type {110}〈112〉 texture with a spread towards a Goss-type {110}〈001〉 texture [3]. On the contrary, as shown in [3,11], the literature results on the texture after recrystallization of the deformed microstructure of different TWIP steels, even of similar compositions, vary from a retained, cold-rolled texture to a weak, randomized one.

Cold rolling, as well as recrystallization, have an impact on the elastic constants; in fact, deformation by cold rolling turns an otherwise isotropic or nearly isotropic polycrystalline solid, defined by only two independent elastic constants, into an orthotropic one, characterized by nine independent constants. Ultrasound has been recognized as a nondestructive technique for inspection and material characterization; the measurement of ultrasound wave velocities has long been used to assess the elastic constants since they quantify a material’s resistance to specific elastic deformations and, as sound is a form of elastic wave that travels in a medium, the stiffness tensor contains information about how these acoustic waves behave [6]. Additionally, since ultrasonic properties are affected by variations in microstructure, such as the crystallographic orientation, grain size and microstructural defect density, monitoring the transmission or reflection of ultrasound provides information about the material structure and its mechanical properties [12,13,14,15,16].

The ultrasonic method described in Section 2 was effectively used in a previous work [17] to monitor the structural evolution of a cold-rolled and recrystallized low-alloy steel. A linear relationship was found between deformation and both birefringence and Poisson’s ratio, and the complex changes in anisotropy produced by the austenization and recrystallization heat treatments were clearly detected. To the best of our knowledge, there is no similar research involving TWIP steels, so the aim of this work was to evaluate the elastic constants of a Fe-0.5 wt% C-21.5 wt% Mn TWIP steel deformed by cold rolling and recrystallized by annealing and assess the evolution of anisotropy. In this way, this ultrasonic method could be used as a nondestructive control tool to optimize cold rolling and annealing of TWIP steels, especially for further mechanical processing, such as deep drawing, which are affected by anisotropy.

## 2. Principles of the Ultrasonic Wave Analysis for the Determination of Elastic Constants

An ultrasonic pulse travelling through a solid generates small elastic stresses and temporary elastic deformations that propagate with finite velocity through the solid; thus, a dynamic equilibrium described by the equations of motion is established. Substitution of the generalized form of Hooke’s law into the equations of motion and consideration of plane harmonic waves propagating in a homogeneous semi-infinite solid medium lead to the Christoffel equation:

(1) $$\left(C_{ijkl}n_{j}n_{k}-\rho V^{2}\delta_{il}\right)u_{i}=0$$

where $C_{ijkl}$ are the second-order elastic constants; $\left(n_{1},n_{2},n_{3}\right)$ are the direction cosines of the normal to the wavefront, indicating, therefore, the direction of propagation of the wave; ρ is the density of the medium, V, the phase velocity; $u_{i}$ is the displacement or polarization vector and $\delta_{ij}$ is the Kronecker delta.

Equation (1) corresponds to three homogeneous equations from which, for every propagation direction considered, three different velocity values arise from the cubic equation in $V^{2}$, obtained by taking the determinant of the coefficient matrix equal to zero. These three values correspond to the phase velocities of three nondispersive ultrasonic waves with mutually perpendicular polarization vectors. Thus, if the elastic constants are known, wave velocities in a material can be predicted by solving the Christoffel equation or, inversely, the elastic constants can be assessed from experimentally measured wave velocities [17].

For an isotropic material, the following relationships are obtained:

(2) $$C_{11}=C_{22}=C_{33}=\rho V_{ii}{}^{2}\equiv \rho V_{L}{}^{2}$$

(3) $$C_{44}=C_{55}=C_{66}=\rho V_{ij}{}^{2}\equiv \rho V_{T}{}^{2}$$

(4) $$C_{12}=\rho \left(V_{ii}{}^{2}-2V_{ij}{}^{2}\right)$$

with $V_{ii}\equiv V_{L}$, the velocity of the longitudinal wave (longitudinally polarized in the direction of propagation *i*), and $V_{ij}\equiv V_{T}$, with *i* ≠ *j*, the velocity of the shear wave (polarized in the *j* direction, transverse to the direction of propagation *i*). Therefore, the values of the elastic constants can be obtained simply by measuring an isotropic material’s density and the velocities of a longitudinal wave and shear wave in any direction of propagation. Since Young’s and shear moduli, as well as Poisson’s ratio, are related to the elastic constants, they can also be calculated from these velocities; in particular, Poisson’s ratio is given by Equation (5) [18]:

(5) $$\nu =\frac{{\left(V_{ii}/V_{ij}\right)}^{2}-2}{2\left[{\left(V_{ii}/V_{ij}\right)}^{2}-1\right]}$$

**Figure 1:**
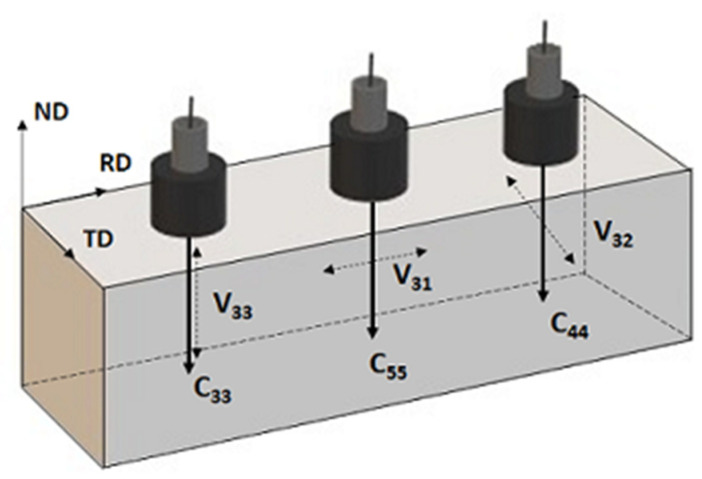
Waves for the determination of elastic constants, with longitudinal and shear waves propagating along ND. The longitudinal wave $V_{33}$ is polarized along the ND axis, while the shear waves of velocities $V_{31}$ and $V_{32}$ are polarized along the RD and TD axes, respectively.

$V_{ii}$ and $V_{ij}$ are independent of their propagation and polarization directions in an isotropic material, so access to any plane is sufficient to calculate their elastic properties. For an orthotropic solid, such as a rolled plate, access to its three planes of symmetry is required to obtain its nine independent constants, $C_{11},\;C_{22},\;C_{33},\;C_{44},\;C_{55},\;C_{66}$ and $C_{12},\;C_{13}\;$ and $C_{23}$. However, to detect variations in the degree of orthotropy of a cold-rolled plate, it is sufficient to measure the velocities of a longitudinal wave and two shear waves propagating through the thickness of the plate, along the ND axis of symmetry (normal to RD, the rolling direction), as shown in Figure 1. The shear waves must be polarized parallel to the RD and TD (transverse to RD) axes of symmetry. In this way, the following relations for $C_{33}$, $C_{44}$ and $C_{55}$ are obtained:

(6) $$C_{33}=\rho V_{33}{}^{2}\;;\;C_{44}=\rho V_{32}{}^{2}\;;\;C_{55}=\rho V_{31}{}^{2}$$

The difference between the elastic constants $C_{44}$ and $C_{55}$ gives rise to the acoustic birefringence (B), which is quantified as the ratio of the difference in velocities $V_{31}$ and $V_{32}$ of the shear waves to their average, as shown by Equation (7), so, for a perfectly isotropic material, B = 0:

(7) $$B=\frac{V_{31}-V_{32}}{\frac{1}{2}\left(V_{31}+V_{32}\right)}=\frac{t_{32}-t_{31}}{\frac{1}{2}\left(t_{31}+t_{32}\right)}$$

where $t_{ij}$ stands for the times of flight of the waves traveling the same path along the ND axis.

Since $V_{31}$ and $V_{32}$ differ in an orthotropic solid, unlike in an isotropic solid, there is no unique value for Poisson’s ratio. Nonetheless, by extrapolating Equation (5) to the orthotropic case, two values for Poisson’s ratio are determined from ultrasound measurements with incidence from the rolling plane; their difference, $\Delta \nu =\nu_{32}-\nu_{31}$, can be used to assess the departure from isotropy [17]:

(8) $$\nu_{31}=\frac{{\left(V_{33}/V_{31}\right)}^{2}-2}{2\left[{\left(V_{33}/V_{31}\right)}^{2}-1\right]}=\frac{{\left(t_{31}/t_{33}\right)}^{2}-2}{2\left[{\left(t_{31}/t_{33}\right)}^{2}-1\right]}$$

(9) $$\nu_{32}=\frac{{\left(V_{33}/V_{32}\right)}^{2}-2}{2\left[{\left(V_{33}/V_{32}\right)}^{2}-1\right]}=\frac{{\left(t_{32}/t_{33}\right)}^{2}-2}{2\left[{\left(t_{32}/t_{33}\right)}^{2}-1\right]}$$

Equations (8) and (9) show that, as with birefringence, Poisson’s ratio requires only the times of flight of the waves, so there is no need to measure the plate thickness.

As pointed out, the solution to the Christoffel equation assumes a homogeneous solid. For inhomogeneous solids, the as-described through thickness measurements will yield average values for the elastic constants, reflecting the effect of the various microstructural characteristics.

## 3. Materials and Methods

A 110 mm long, 150 mm wide and 20.7 mm thick TWIP steel ingot was homogenized at 1000 °C for one hour, hot rolled to a 50% reduction and subsequently cooled in air. Its chemical composition is given in Table 1.

Sixteen samples of dimensions 7.3 mm × 23 mm × 55 mm were cut for cold rolling, which were grouped into 3 sets of 5 specimens each and one as a sample pattern. In each set, the cold-rolling process was carried out to thickness reductions of 6, 16, 31, 52 and 70%.

Each set of cold-rolled samples underwent an annealing heat treatment in a furnace model 56667-E (Lindberg, Buenos Aires, Argentina). Table 2 indicates the treatment temperatures and times. In all cases, once the treatment was finished, the specimens were cooled in water to freeze their structure.

To obtain ultrasonic velocities, elastic constants and birefringence, ultrasonic tests were carried out before and after each reduction and after each heat treatment. The ultrasonic system consisted of a 5077PR transmitter–receiver (Panametrics, Worcester, MA, USA) in pulse-echo mode; two 5 MHz, 11 mm diameter Panametrics contact transducers (V460 for longitudinal waves and V155 for shear waves) and an HS805 oscilloscope (TiePie, Sneek, The Netherlands), used to obtain and store the signals. A suitable coupler was used for each type of transmitted wave. The times of flight were measured as the peak-to-peak times between the first two consecutive echoes.

The surfaces of all the specimens were prepared to couple the longitudinal and shear transducers. Two zones were selected and identified on the top surface of each sample (RD–TD plane) to measure the thickness with a micrometer and adequately propagate the ultrasonic longitudinal and shear waves along the thickness direction. Thus, a total of six measurements were performed for each reduction.

To relate the results to standard techniques, metallographic samples were prepared using standard polishing procedures, over-etching with 3% nital for 5 s, followed by 3 s polishing with alumina 3 to partially remove the over-etching. The micro-indentation Vickers hardness with a 250 gf test force was measured before and after deforming the samples and after each heat treatment.

## 4. Results and Discussion

### 4.1. Cold Rolling

#### 4.1.1. Effect of Cold Rolling on Microstructure and Hardness

**Figure 2:**
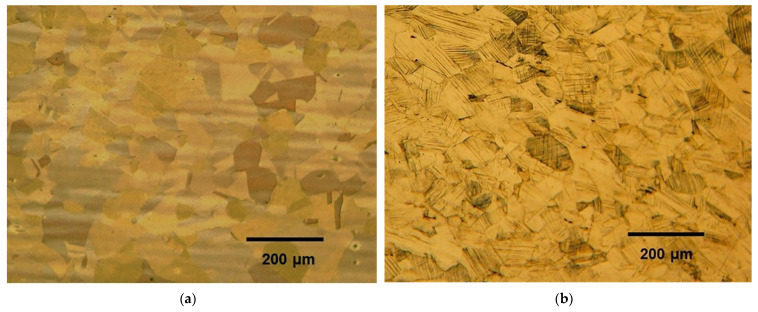

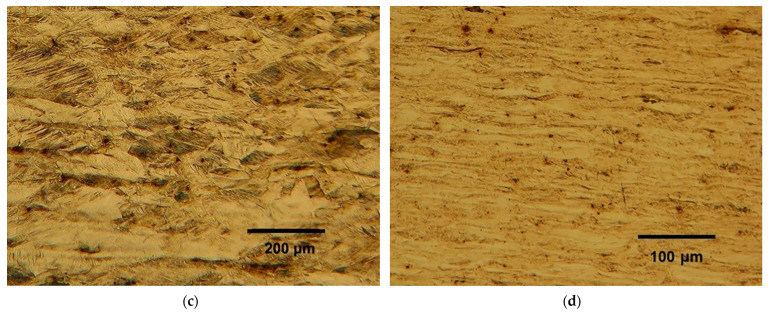
Microstructures of the Fe-0.5 C-21.5 Mn TWIP steel, cold rolled to reductions of (**a**) 0%, (**b**) 6%, (**c**) 31% and (**d**) 52%.

Figure 2a shows the full austenitic microstructure of the TWIP steel hot rolled at 1000 °C with no cold reduction. The microstructures obtained after cold rolling to a 6% reduction (Figure 2b) show the early presence of strain marks, which are mainly attributed to slip bands and, to a lesser extent, to deformation twins for this reduction stage [19,20]. As reduction increased up to 52%, the number of marks increased in most of the grains, which are elongated in shape due to the extensive reduction, and the twin-lamellar structure tends to follow the rolling direction (Figure 2c,d). This rotation leads to further slip difficulty since the Schmid factor for slip on either the twin planes or the {111} planes decreases greatly [20]. Duggan et al. [21] explained that for further deformation to occur, an instability must develop, which, in this case, is a shear band or cutting band that circumvents the restrictive conditions of geometric alignment and orientation of the structure. At a 70% reduction, the structure is highly textured and resistant to any normal processes of crystallographic deformation, be it homogeneous sliding or twinning; hence, a condition of high elastic stress prevails, with the formation of profuse shear bands.

**Figure 3:**
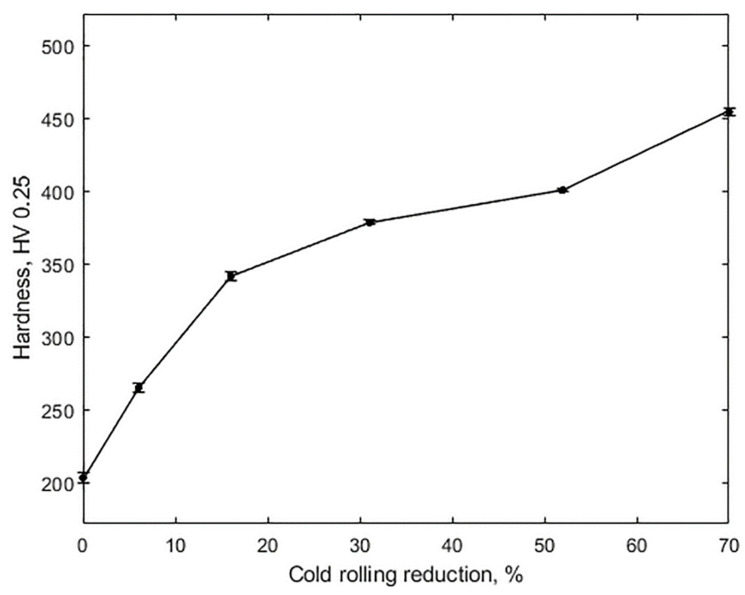
Hardness vs. thickness reduction by cold rolling.

Three distinct stages in hardness are apparent in Figure 3, where the hardness curve shows a steep slope up to a 16% reduction, with the hardness increasing from 201 to 344 HV, followed by a marked decrease between 16 and 52% and a final increase to 453 HV at 70%. This variable slope is closely associated with the prevailing deformation mechanisms described above. In fact, in the work of Haase et al. [22], who calculated the twinned volume percentage in all grains of a TWIP steel of similar composition to the one studied in this work, it was reported that the twinned volume fraction began to increase at nearly 15% reduction by cold rolling, reaching saturation at about 50%. Thus, the decrease in the slope in the 16–52% reduction range might be related to the increase in the percentage of twinning until saturation.

#### 4.1.2. Effect of Cold Rolling on Wave Velocities and Elastic Constants

**Figure 4:**
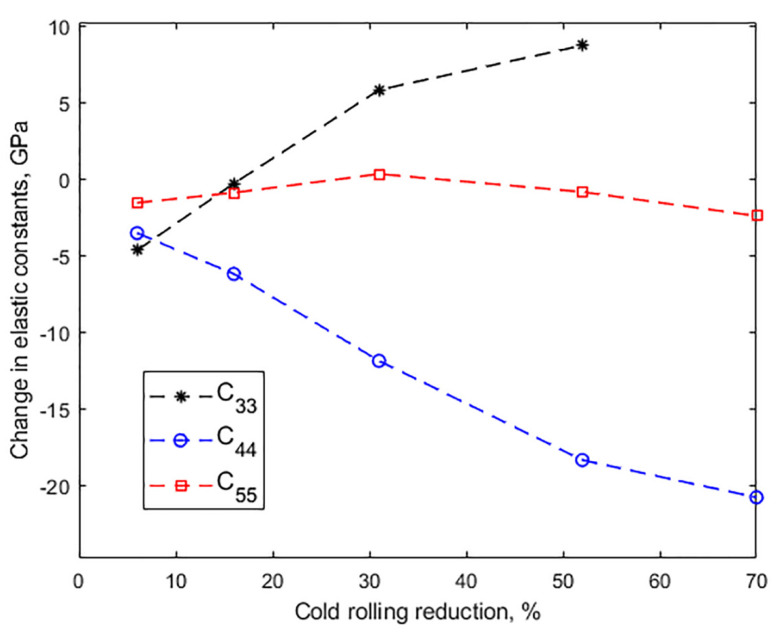
Effect of the thickness reduction by cold rolling on the change in elastic constants.

Table 3 relates the measured ultrasonic velocities and the corresponding values of the elastic constants to reduction by cold rolling. From these data, Figure 4 shows the changes in the elastic constants from their original values.

Initially, at a 6% reduction, due to the interaction of the waves with the microstructural defects and deformation mechanisms produced during the first stages of rolling, such as glide dislocation [23], the values of the three velocities and consequently of the three elastic constants decreased. From there on, however, $V_{33}$ increased steadily by up to 1.8% (which implies a difference of 8.7 GPa in $C_{33}$) when reduction reached 52%, while $V_{31}$ recovered at 31% reduction to decrease by 1.7% at 70% reduction; that is a difference of only 2.4 GPa in $C_{55}$. The effect on $V_{32}$—that is, on the velocity of the shear wave perpendicularly polarized to the rolling direction—, and consequently, on $C_{44}$, as shown in Figure 4, was remarkably stronger. In fact, as the reduction increased to 52%, the velocity decreased by 411 ms^−1^, or 13.4%. A qualitatively similar behavior was observed in a low-alloy steel [17]; there, however, $V_{32}$ only decreased 2.7% at a 52% reduction, so, for the TWIP steel, the twin-lamellar structure that follows the rolling direction, as described above, must play a role in such a prominent effect. Ultimately, at a 70% reduction, with the presence of profuse shear bands, the velocity decreased even further, totaling 471 ms^−1^; that is a fall of more than 22 GPa or 28% in $C_{44}$.

#### 4.1.3. Effect of Cold Rolling on Poisson’s Ratio and Birefringence

**Figure 5:**
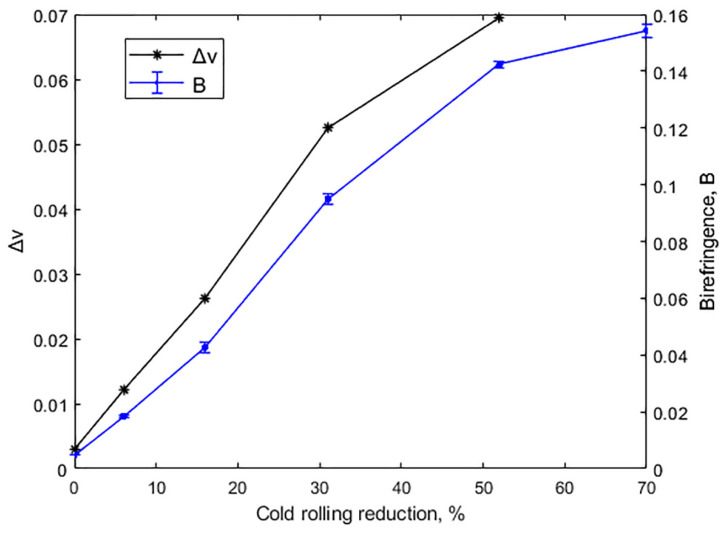
Acoustic birefringence (B) and difference in Poisson’s ratio ($\Delta \nu =\nu_{32}-\nu_{31}$ ) vs. thickness reduction by cold rolling.

Acoustic birefringence (B) and Poisson’s ratios ($\nu_{32}$ and $\nu_{31}$) are shown in Table 4 as a function of reduction. Since Poisson’s ratio requires knowledge of the longitudinal wave time of flight, it could not be calculated for a 70% reduction. It is seen that while $\;\nu_{31}$, which depends on $V_{31}$, grew 6% at a 52% reduction, $\nu_{32}$ increased 30% due to the highlighted effect of cold rolling on $V_{32}$. B, on the other hand, varied between 0.005 without cold reduction to 0.154 at 70%, and, as Figure 5 shows, the greatest increase occurred between 16 and 52% reduction. Δν, the difference between Poisson’s ratios $\nu_{32}$ and $\nu_{31}$, also shown in Figure 5, followed a similar trend. That is, the onset of orthotropy as cold rolling proceeded, which in terms of texture was related by Bouaziz O. et al. [4] to the presence of three components (Goss, brass and copper) for a 50% reduction, is clearly reflected by birefringence and Δν. Additionally, the three different stages described in Figure 3 are apparent here.

### 4.2. Annealing

#### 4.2.1. Effect of Annealing on Microstructure and Hardness

**Figure 6:**
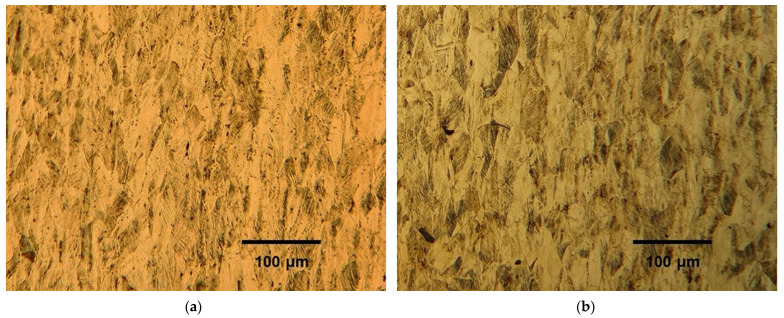

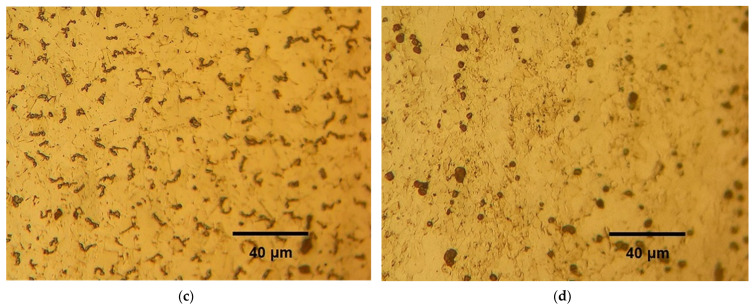
Microstructures of the Fe-0.5 C-21.5 Mn TWIP steel cold rolled to 31% (**a**,**b**) and 52% (**c**,**d**), after annealing at 600 °C (**left**) and 700 °C (**right**).

Although optical microscopy does not allow for the quantification of the decrease in strain marks such as twins, the effect of annealing can still be visualized (Figure 6). The microstructures obtained after annealing show that the strain marks produced by cold rolling were still present in the samples rolled to 31% reduction and annealed at 600 °C for 30 min (Figure 6a) and 700 °C for 5 min (Figure 6b). In the sample cold rolled to 52% reduction and annealed at 600 °C for 30 min (Figure 6c), the nucleation of new grains is perceived, and in the one annealed 5 min at 700 °C (Figure 6d), the microstructure shows an extremely fine nucleation wherein the presence of strain marks is no longer visible.

**Figure 7:**
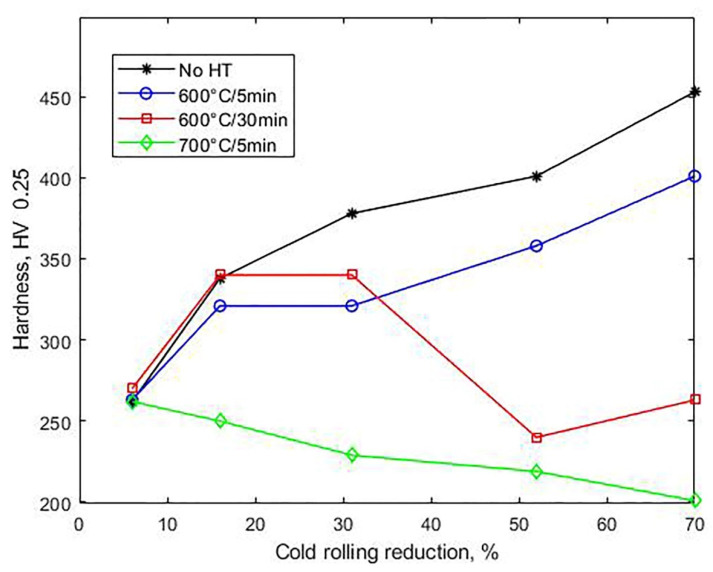
Effect of recrystallization annealing after cold rolling on Vickers hardness. As a reference, data for specimens without heat treatment (No HT) are also shown.

Figure 7 shows that none of the annealing treatments used induced a significant change in hardness at a 6% reduction. For higher strains, increasing the temperature from 600 °C to 700 °C at a fixed soaking time of 5 min clearly meant a higher drop in hardness. While at 70% reduction, annealing 5 min at 700 °C returned hardness to its value before cold rolling (201 HV 0.25), even a higher annealing time, 30 min, at 600 °C was not enough to do so. This behavior can be explained from the graphs obtained by Ferraiuolo et al. [24], which show that a sample deformed at 60% and annealed at 600 °C recrystallizes with negligible grain growth, while at 700 °C, recrystallization is complete in 3 min. Figure 7 also shows that for reductions of 16 and 31%, there was a slight increase in hardness when the time at 600 °C increased from 5 to 30 min. This may be due to carbide precipitation, such as (Fe,Mn)_3_C.

#### 4.2.2. Effect of Annealing on Wave Velocities and Elastic Constants

Table 5 and Table 6 show the ultrasonic velocities and corresponding elastic constants after annealing the cold-rolled samples. When compared with the values in the cold-rolled condition, the data show that up to a 16% reduction, changes in the elastic constants induced by heat treating at 600 °C were minimal (less than 1 GPa), which in some cases lie within the standard deviation. This also holds true for the samples annealed at 700 °C after cold rolling to a 6% reduction.

**Figure 8:**
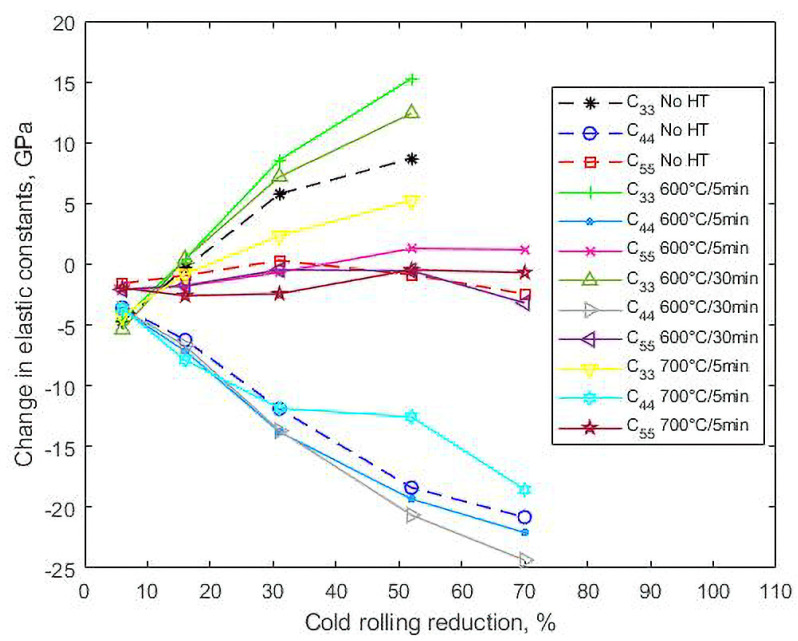
Cold rolling and annealing-induced shift in C_33_, C_44_ and C_55_ from their original values before cold rolling. As reference, data for specimens without heat treatment (dash lines, No HT) are also shown.

At higher reductions, in general, annealing at 600 °C shifted the values of the constants away from the as-rolled condition, and, consequently, farther from the original values. This is best seen in Figure 8, which shows the shift induced by both cold rolling and annealing in $C_{33}$, $C_{44}$ and $C_{55}$ from their original values before cold rolling. The shift induced by cold rolling alone (see Figure 4) is shown as the baseline. For $C_{33}$, a greater shift in the samples annealed for 5 min is observed, which is seen to decrease by about half after annealing for 30 min. A similar effect of time occurred on $C_{55}$ at the highest strains. On the contrary, the shift in $C_{44}$ is seen to increase with time at 600 °C. Finally, Figure 8 shows that as the annealing temperature was increased to 700 °C, the values of the constants, especially for the higher strains, shifted away from the cold-rolled values towards the original strain-free values, although significant differences remain. In fact, at a 52% reduction, these differences with the original values are 5.2 GPa and 12.6 GPa in $C_{33}$ and $C_{44}$, respectively.

#### 4.2.3. Effect of Annealing on Poisson’s Ratio and Birefringence

**Figure 9:**
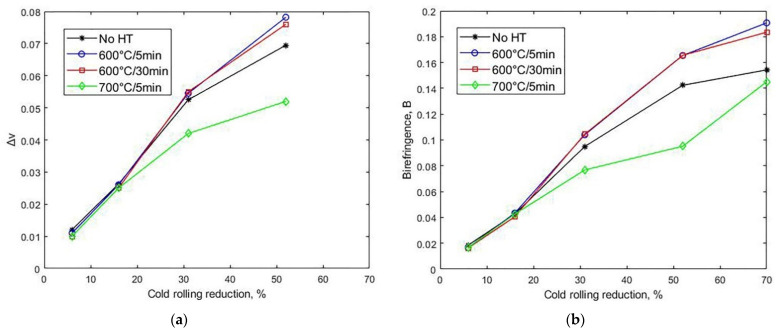
Effect of recrystallization annealing after cold rolling on (**a**) difference in Poisson’s ratio ($\Delta \nu =\nu_{32}-\nu_{31}$) and (**b**) acoustic birefringence (B). As reference, data for specimens without heat treatment (No HT) are also shown.

The somewhat complex behavior of the elastic constants after annealing are better understood by analyzing the difference in Poisson’s ratio ($\Delta \nu =\nu_{32}-\nu_{31}$) and birefringence, recalling that they reflect the difference in $C_{44}$ and $C_{55}$ and which, as mentioned, have the additional advantage of being functions only of the time of flight of the waves. Figure 9 shows that, in line with the behavior of the elastic constants, annealing of samples cold rolled up to 16% did not change the anisotropy acquired by cold rolling. For higher reductions, annealing at 600 °C increased both B and Δν, and increasing the annealing time at a 52% reduction slightly decreased Δν due to the fall in $C_{33}$. On the other hand, the decrease in B with annealing time at a 70% reduction is due to the relative displacement of $C_{44}$ and $C_{55}$. At 700 °C, although both B and Δν decreased from the cold-rolled values, with the greatest drop occurring at 52%, they are still much higher than in the hot-rolled state, where Δν=0.003 and B=0.005.

The described effects on the elastic constants and, consequently, on birefringence and Δν mean that orthotropy is retained after the recrystallization anneals. These results are consistent with those in the work by Bracke et al. [11], who studied the texture generation after recrystallization annealing of a TWIP steel of similar composition to the one studied here. They proved their hypothesis that recrystallization occurs in the absence of significant recovery, from an energetically relatively homogenous deformed microstructure, which leads to a recrystallization mechanism described as site-saturated nucleation with random-orientation sampling from the cold-rolled texture. Thus, the rolling texture is retained since no specific orientations of the deformed matrix have an energetic advantage.

From the preceding discussions, while hardness values after annealing the previously cold-rolled TWIP steel may show that there is partial or total recrystallization, the elastic constants and especially Δν and birefringence clearly evidence the effect of the recrystallization process on anisotropy. Thus, although these elastic parameters bear no information on the type of texture, they do tell whether the recrystallization texture is strong, weak or randomized, so this nondestructive ultrasonic evaluation may be used as a fast and simple tool to control the production of cold-rolled and annealed TWIP steels, as well as assist in the development of processes aiming to obtain texture-free TWIP steels.

## 5. Conclusions

Measurements of the time of flight and velocity of ultrasonic waves in a Fe-0.5 C-22 Mn TWIP steel allowed us to follow the changes induced by cold rolling and recrystallization annealing in its elastic constants, $C_{33}$, $C_{44}$ and $C_{55}$. Poisson’s ratio and acoustic birefringence were determined and used as parameters to assess anisotropy. The main conclusions are summarized as:

- Increasing reduction by cold rolling markedly decreased $C_{44}$, had a small effect on $C_{55}$ and increased $C_{33}$, thus increasing the orthotropy, which was reflected in the values of birefringence and the Poisson’s ratio parameter Δν.
- The curve of hardness versus reduction by cold rolling shows a variable slope, which may be related to the deformation mechanisms. This variable slope is also present in the birefringence and Δν curves.
- The effects of the recrystallization annealing temperature and time were reflected in both hardness and the elastic parameters. While the former shows that for medium to high deformation, there was substantial recrystallization at both 600 °C and 700 °C, the latter show that orthotropy was retained after recrystallization.

## Figures and Tables

**Table 1:** TWIP steel chemical composition, wt% *.

C (%)	Si (%)	Mn (%)	Al (%)	N (%)
0.51	0.296	21.47	0.0056	0.052

* Measured by optical emission spectrometry.

**Table 2:** Time and temperature for heat treatment of cold-rolled samples.

	Set 1	Set 2	Set 3
**Temperature, °C**	600	600	700
**Time, min**	5	30	5

**Table 3:** Wave velocities and elastic constants vs. reduction by cold rolling.

Reduction (%)	Wave Velocities (m/s); Elastic Constants (GPa)		
	$V_{33}$ $C_{33}$	$V_{32}$ $C_{44}$	$V_{31}$ $C_{55}$
**0**	5468.01 ± 0.00 233.81 ± 0.00	3065.38 ± 0.00 73.46 ± 0.00	3079.42 ± 0.00 74.14 ± 0.00
**6**	5412.82 ± 5.30 229.12 ± 0.45	2989.81 ± 5.77 69.90 ± 0.27	3045.63 ± 6.93 72.56 ± 0.33
**16**	5464.30 ± 6.19 233.49 ± 0.53	2932.18 ± 6.25 67.23 ± 0.29	3060.16 ± 3.46 73.22 ± 0.17
**31**	5534.96 ± 3.85 239.6 ± 0.33	2805.71 ± 8.33 61.56 ± 0.37	3085.42 ± 6.11 74.44 ± 0.30
**52**	5568.69 ± 13.43 242.5 ± 1.17	2654.11 ± 4.58 55.08 ± 0.19	3061.00 ± 6.08 73.27 ± 0.29
**70**	*	2594.37 ± 4.51 52.65 ± 0.18	3028.12 ± 4.36 71.70 ± 0.21

* The calculated wavelength of the 5 MHz longitudinal wave approaches half the sample thickness when cold rolled to 70% (2.15 mm), which precluded measuring its time of flight.

**Table 4:** Birefringence and Poisson’s ratio vs. reduction by cold rolling.

Reduction (%)	B	$\nu_{32}$	$\nu_{31}$
**0**	0.005 ± 0.000	0.271 ± 0.000	0.268 ± 0.000
**6**	0.018 ± 0.000	0.280 ± 0.002	0.268 ± 0.002
**16**	0.043 ± 0.002	0.298 ± 0.001	0.272 ± 0.000
**31**	0.095 ± 0.002	0.327 ± 0.001	0.275 ± 0.000
**52**	0.142 ± 0.001	0.353 ± 0.001	0.284 ± 0.001
**70**	0.154 ± 0.002	*	*

* Note: Since Poisson’s ratio requires knowledge of the longitudinal wave time of flight, there are no data for 70% reduction.

**Table 5:** Wave velocities after recrystallization annealing cold-rolled specimens.

Reduction (%)	Wave Velocity (m/s)								
	600 °C/5 min			600 °C/30 min			700 °C/5 min		
	$V_{33}$	$V_{32}$	$V_{31}$	$V_{33}$	$V_{32}$	$V_{31}$	$V_{33}$	$V_{32}$	$V_{31}$
**6**	5414.00 ± 4.24	2984.69 ± 8.54	3034.69 ± 8.01	5405.70 ± 0.99	2986.72 ± 3.24	3035.71 ± 0.00	5418.17 ± 4.00	2988.33 ± 3.11	3038.17 ± 3.64
**16**	5473.00 ± 15.55	2912.09 ± 10.32	3041.28 ± 16.81	5473.18 ± 1.66	2921,51 ± 3.00	3042.38 ± 0.00	5458.38 ± 6.18	2895.43 ± 2.89	3024.77 ± 3.53
**31**	5567.42 ± 17.55	2762.07 ± 4.33	3065.13 ± 15.34	5551.36 ± 22.12	2764.44 ± 16.88	3069.53 ± 13.70	5495.31 ± 4.68	2806.92 ± 18.50	3028.11 ± 11.44
**52**	5643.70 ± 2.55	2631.20 ± 11.57	3105.79 ± 15.46	5611.20 ± 16.80	2598.82 ± 2.51	3067.90 ± 15.86	5531.40 ± 8.71	2790.75 ± 4.31	3069.52 ± 15.87
**70**		2563.31 ± 13.55	3103.70 ± 4.10		2505.94 ± 2.10	3012.16 ± 10.23		2650.03 ± 27.20	3064.53 ± 29.81

**Table 6:** Elastic constants after recrystallization annealing cold-rolled specimens.

Reduction (%)	Elastic Constants (GPa)								
	600 °C/5 min			600 °C/30 min			700 °C/5 min		
	$C_{33}$	$C_{44}$	$C_{55}$	$C_{33}$	$C_{44}$	$C_{55}$	$C_{33}$	$C_{44}$	$C_{55}$
**6**	229.22 ± 0.36	69.66 ± 0.40	72.02 ± 0.38	228.43 ± 0.08	69.76 ± 0.15	72.07 ± 0.00	229.57 ± 0.34	69.83 ± 0.15	72.18 ± 0.17
**16**	234.24 ± 1.33	66.32 ± 0.47	72.33 ± 0.80	234.25 ± 0.14	66.75 ± 0.14	72.38 ± 0.00	232.99 ± 0.53	65.56 ± 0.13	71.55 ± 0.17
**31**	242.39 ± 1.53	59.66 ± 0.19	73.47 ± 0.74	241.00 ± 1.92	59.76 ± 0.73	73.68 ± 0.66	236.15 ± 0.40	61.61 ± 0.81	71.71 ± 0.54
**52**	249.08 ± 0.23	54.14 ± 0.48	75.43 ± 0.75	246.22 ± 1.48	52.82 ± 0.10	73.60 ± 0.76	239.05 ± 0.75	60.90 ± 0.19	73.68 ± 0.76
**70**		51.38 ± 0.54	75.33 ± 0.20		49.11 ± 0.08	70.95 ± 0.48		54.92 ± 1.13	73.45 ± 1.43

## Data Availability

All data are contained within the article.
